# Supplementary material for: Learning curve and surgical outcome of robotic assisted colorectal surgery with ERAS program
Source: Sci Rep. 2022 Nov 29;12:20566. doi: 10.1038/s41598-022-24665-w (PMC9709162; doi:10.1038/s41598-022-24665-w)
Supplement: Supplementary file 2 — Supplementary Information 2. [file 41598_2022_24665_MOESM2_ESM.zip › RAL Raw data-Ñ[▒K/125/HS_ñw╝╨░O▒Kñσ.pdf]

正式摘要(電子簽章：000000230098422)

W52 / W52 ☐ 英文標題(注意:無英文姓名-請補病人基本檔!) ☐【讀取】憑證

臺中榮民總醫院

出院病歷摘要

轉入號 : 轉出號 :

[redacted]

(8)流水編號	
(9)入院日期	110/11/12 (2021) 直腸外科 W52 - 161
(10)轉科日期	
(11)出院日期	110/11/17 (2021) 住院天數計 5 日

診斷

- 入院 :1. Rectum adenocarcinoma, FAV 2cm,
  - s/p Short course neoadjuvant CCRT with Ufur and Tomotherapy 25Gy/5fr,
  2. HTN
- 出院 :1. Rectum adenocarcinoma, FAV 2cm,
  - s/p Short course neoadjuvant CCRT with Ufur and Tomotherapy 25Gy/5fr,
  - s/p Robotic assisted LAR + loop-ileostomy on 2021/11/13
  2. HTN

主訴

- Anal bleeding for 2 years

病史

- This is a 47 years old male patient with history of

  1. HTN

  According to the medical record, he had bloody stool for 2 years. He visit Fong-Yuan H. where colonoscopy with biopsy was done, showed Adenocarcinoma, FAV 2cm. Thus, he came to our CRS OPD for second opinion. Then, abdominal CT was arranged on 2021/11/02, showed Rectum cancer. Short course neoadjuvant CCRT with Ufur and Tomotherapy 25Gy/5fr. This time, he was admitted to our ward for scheduled Robotic assisted LAR.

體檢發現

-
  - General appearance :
    - easy looking , conscious clear , without cardiopulmonary distress
    - Vital signs自動帶入date 體溫(BT) 脈搏(P) 呼吸(R) 血壓(BP) 身高(BH) 體重(BW) BMI
      20211112 36.3℃ 78次/分 18次/分 131/68mmHg 155cm 57.9kg 24.1
    - Vital signs: B.P. 131/68 mmHg , P.R. 78 /min , R.R. 18 /min , B.T. 36.3 ℃
  - HEENT :
    - Eyes: no anemic conjunctiva, no icteric sclera, normal EOM
    - ENT: no otorrhea, no rhinorrhea, no erythematous mucosa, no injected tonsil, no deformity
  - Neck: supple, freely movable, trachea midline,no deformity, no engorged jugular vein , no palpable thyroid or lymph node enlargement
  - Chest:
    - Inspection: Grossly normal, symmetric and fully expansion, no accessory muscle used, no operation scar, no spider angiomata, no gynecomastia
    - Palpation: no palpable mass or nodule, no subcutaneous emphysema, no tenderness
    - Ausculation: clear breathing sound
  - Heart:
    - Palpation: no thrill, no heave
    - Percussion: normal heart size
    - Ausculation: regular heart beat, normal S1, S2, no gallop, no murmur
  - Extremities: No deformity, full ranged of motion without limitation, no clubbing finger or

- nail cyanosis, no pitting edema, no ulcer or pigmentation, no obvious skin lesion, no varicose vein, no bone, joint or muscle tenderness

CRS CONDITION

- Karnofsky performance status: (100) Normal, no complaints, no evidence of disease
- PE of Abdomen:
- CEA: . ng/ml
- Imaging Studies:
- Colonoscope report: rectum ca, 2cm FAV
- Figures:
    1. colon & rectum

手術日期及方法

- 手術日期及方法20211113 1. Robotic (XI) assisted TME
  2. Intraoperative colonoscopy : (-).
  3. High ligation (+) : At IMA root (Via robotic clips). IMV was ligated via endoclip.
  4. Take down from the splenic flexure : (-)
  5. Robotic EndoGIA (45-4.3) X2 + EEA (29mm) + OpenGIA (80-3.8) X1 were applied to complete the side-to-end anastomosis
  6. Proximal cut end: > 10 cm, distal cut end: 5 cm
  7. No drainage tube was inserted
  8. ADHESION BARRIER FILM was placed
  9. Loop ileostomy (About 15~20cm from IC valve) was done over RLQ of abdomen

住院治療經過

- After admission, he recieved Robotic assisted LAR + loop-ileostomy on 2021/11/13. We remove the NG tube on 11/14 and let him try bland I diet. CVC was removed on 11/16 and try bland II diet. Under relative stable condition, he was discharged on 11/16 and OPD f/u.

合併症

一般檢查

- 累積報告 :

DATE	TIME	ALB	TP	BIL.T	BIL.D	ALKP	AST	ALT	LDH	AMY	LIPASE	NH3	Ferritin	UA
1101112	1707	4.6	7.0	0.3	0.1	84	15	20						6.4
1101113	1522	3.9		0.64				17						

DATE	TIME	WBC	RBC	HGB	HCT	MCV	PLT	BLST	NEUT	BAND	SEG	LYM	MON	EOS	BAS
1101112	1705	7770	5.25	15.8	45.2	86.1	221		73.8			15.2	6.0	4.5	0.5
1101113	1519	14610		14.2	42.0		189		91.8			4.4	3.1	0.5	0.2

DATE	TIME	NA	K	CL	BUN	CREAT	eGFR	CA	Mg	P	CRP	ESR	PCT	D-Dimer	LACTATE
1101112	1707	139	3.8		15	0.94	91.43	9.2			0.065				
1101113	1522	140	3.6	105	11	0.86	101.31	8.4							

DATE	TIME	GLUCOSE	HbA1C	CHOL	TG	HDL	LDL	KETONE	CK	CKMB	TRO-T	PT-P	APTT-P	NT-pro BNP
1101112	1705	91										10.6	29.8	
1101112	1707			160	170	41	96							

特殊檢查

病理報告

其它

出院時情況

- 3.改門診治療

出院指示

-

藥名	劑量	劑量單位	用法	頻次	數量	數量單位	現況	天數	單位
(口)Takepron OD tab 30mg	1	TAB	PO	QDAC	7	TAB	TH	7	天
Ultracet tab	1	TAB	PO	Q6H	28	TAB	TH	7	天
Celebrex cap 200mg	1	CAP	PO	BID	14	CAP	TH	7	天
OCAS Harnalidge 0.4 mg	1	TAB	PO	QDAC	7	TAB	TH	7	天

出院計劃

- 門診日期
    - date on 2021/11/24

主治醫師 2062F 蔣鋒帆 / 住院醫師 2339C 蔡濬樟

正式摘要(電子簽章：000000230098422)

W52 / W52

doctorRank:B
